# CCN1 promotes hepatic steatosis and inflammation in non-alcoholic steatohepatitis

**DOI:** 10.1038/s41598-020-60138-8

**Published:** 2020-02-21

**Authors:** Linling Ju, Yan Sun, Hong Xue, Lin Chen, Chunyan Gu, Jianguo Shao, Rujian Lu, Xi Luo, Jue Wei, Xiong Ma, Zhaolian Bian

**Affiliations:** 1 Nantong Institute of Liver Disease, Department of Gastroenterology and Hepatology, Nantong Third People’s Hospital, Nantong University, Nantong, Jiangsu China; 2 Department of Neurology, Affiliated Hospital of Nantong University, Nantong, Jiangsu China; 3 Liver Diseases Infectious Diseases, Nantong Third People’s Hospital, Nantong University, Nantong, Jiangsu China; 4 Department of Pathology, Nantong Third People’s Hospital, Nantong University, Nantong, Jiangsu China; 5 Department of Cardiothoracic Surgery, Nantong Third People’s Hospital, Nantong University, Nantong, Jiangsu China; 6 Department of Gastroenterology, Tongren Hospital, Shanghai Jiaotong University School of Medicine, Shanghai, China; 7 State Key Laboratory for Oncogenes and Related Genes, Key Laboratory of Gastroenterology & Hepatology, Ministry of Health, Division of Gastroenterology and Hepatology, Ren Ji Hospital, School of Medicine, Shanghai Jiao Tong University, Shanghai Cancer Institute, Shanghai Institute of Digestive Disease, Shanghai, China

**Keywords:** Gastrointestinal cancer, Gastrointestinal diseases

## Abstract

Non-alcoholic fatty liver disease (NAFLD) is characterized by increased uptake and accumulation of lipids in hepatocytes. Simple steatosis may progress to non-alcoholic steatohepatitis (NASH) with inflammation, hepatocellular injury and fibrosis. CCN1 is an important matrix protein that regulates cell death and promotes immune cell adhesion and may potentially control this process. The role of CCN1 in NASH remains unclear. We investigated the role of CCN1 in the pathogenesis of steatohepatitis. CCN1 upregulation was found to be closely related with steatosis in patients with NASH, obese mice and a FFA-treated hepatocyte model. Controlling the expression of CCN1 in murine NASH models demonstrated that CCN1 increased the severity of steatosis and inflammation. From the sequence results, we found that fatty acid metabolism genes were primarily involved in the MCD mice overexpressing CCN1 compared to the control. Then, the expression of fatty acid metabolism genes was determined using a custom-designed pathway-focused qPCR-based gene expression array. Expression analysis showed that CCN1 overexpression significantly upregulated the expression of fatty acid metabolism-associated genes. *In vitro* analysis revealed that CCN1 increased the intracellular TG content, the pro-inflammatory cytokines and the expression level of apoptosis-associated proteins in a steatosis model using murine primary hepatocytes. We identified CCN1 as an important positive regulator in NASH.

## Introduction

Non-alcoholic fatty liver disease (NAFLD) has been identified as an increasing public health burden^1^. NASH, the more severe form of NAFLD, is characterized by hepatocellular ballooning, steatosis and lobular inflammation in liver histology^1–3^. NASH may not only progress to advanced hepatic fibrosis and cirrhosis, even hepatocellular carcinoma, but also significantly increases the risk of other diseases, such as cardiovascular disease and diabetes. Inflammation in the liver plays a critical role in the development of NASH, as well as in the induction and progression of liver fibrosis. However, the molecular mechanisms that lead to progression from isolated steatosis to NASH have not been completely elucidated. Recent studies have shown that many genes involved in fatty acid metabolism, insulin signalling pathways, inflammatory pathways, oxidative stress and fibrogenesis play a role in NAFLD/NASH pathogenesis^4–7^.

The CCN family, including cysteine-rich protein 61 (Cyr61, also known as CCN1), connective tissue growth factor, and nephroblastoma overexpressed, are matrix-associated proteins that play critical roles in growth, differentiation, angiogenesis, migration, and extracellular matrix regulation^8–10^. CCN1 is a secreted, heparin-binding protein and is involved in a variety of diseases, including carcinoma^11^, inflammation^12^, fibrosis^13,14^, and autoimmune disease^15^. In the liver, it has been shown to be a tumour suppressor^16^ and is involved in the oxidative DNA damage response in concanavalin A (ConA)-treated livers^17^. Recently, we reported that CCN1 induced by FFAs in hepatocytes results in macrophage infiltration and hepatic inflammation in a NASH model^18^. It is generally assumed that steatosis is the initiation factor in the pathogenesis of NAFLD^19^. We investigated the role of CCN1 in the pathogenesis of steatosis in a murine and cellular model. In our study, we found that CCN1 was correlated with steatosis in patients with NASH. We conclude that increased CCN1 may act as an initiation factor in steatosis in NASH.

## Results

### CCN1 expression was upregulated in patients with NASH and mouse models

**Figure 1:**
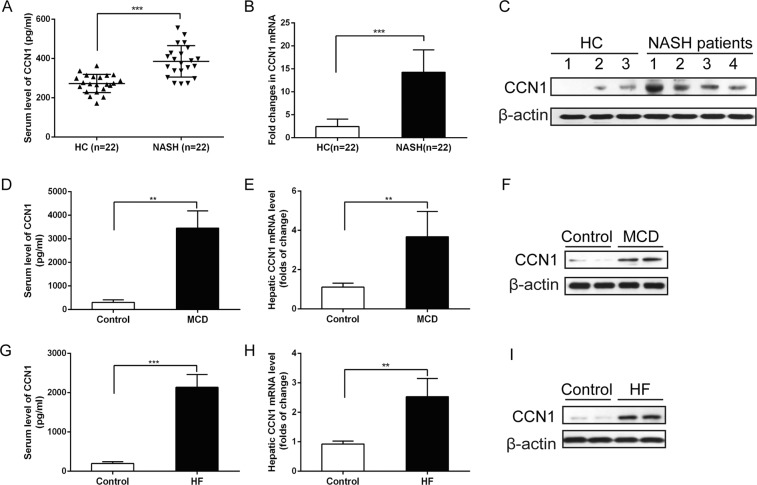
The levels of CCN1 were increased in patients with NASH and mouse models. (**A**) Serum CCN1 levels were increased in patients with NASH (369.8 ± 90.1 pg/ml, p < 0.01) compared with healthy controls (240.4 ± 42.1 pg/ml); (**B**). Hepatic CCN1 mRNA detected by qPCR in patients with NASH and healthy controls; (**C**). Hepatic CCN1 protein detected by western blot in patients with NASH and healthy controls; (**D**). Serum level of CCN1 in MCD mice and controls; (**E**). Hepatic CCN1 mRNA detected by qPCR in MCD mice and controls; (**F**). Hepatic CCN1 protein detected by western blot in MCD mice and controls; (**G**). Serum level of CCN1 in HF mice and controls; H. Hepatic CCN1 mRNA detected by qPCR in HF mice and controls; I. Hepatic CCN1 protein detected by western blot in HF mice and controls; Statistical significance was assessed by two-tailed Student’s t test. The error bar indicates the SD. **P < 0.01; ***P < 0.001.

The main clinical and biochemical features of patients with NASH and healthy controls are shown in Table 1. There were no statistically significant differences in sex or age between the two groups. Body mass index (BMI), serum alanine aminotransferase (ALT), aspartate aminotransferase (AST) and gamma glutamyl transpeptidase (GGT) levels were higher in the patients with NASH than in the healthy controls. To investigate whether CCN1 expression was altered in patients with NASH, we detected its expression level by ELISA in the serum of patients with NASH (n = 22) and healthy controls (HC, n = 22). Serum CCN1 protein levels were higher in patients with NASH (369.8 ± 90.1 pg/ml) than in healthy controls (240.4 ± 42.1 pg/ml) (Fig. 1A). Moreover, the levels of hepatic CCN1 were detected in patients with NASH and healthy controls by real time (RT)-PCR and western blotting. The levels of hepatic CCN1 mRNA and protein in patients with NASH were significantly increased compared with those in healthy controls (p < 0.01) (Fig. 1B,C). In addition, we analysed CCN1 expression in different murine models of NAFLD, namely, mice fed a methionine choline deficient (MCD) or a high-fat (HF) diet, and found that CCN1 expression was dramatically increased in mice fed an MCD or HF diet compared with mice fed chow diets in serum and liver (Fig. 1D–I).

**Table 1:** Clinical and biochemical features of healthy controls and patients with NASH.

Factors	Healthy controls (n = 22)	NASH (n = 22)
Female (n)	14	11
Age (years)	50.30 ± 7.96	51.10 ± 11.47
BMI (kg/m^2^)	21.6 ± 4.2	27.3 ± 5.1**
ALT (UI/I)	21.60 ± 10.37	132.70 ± 552.0**
AST (UI/I)	18.00 ± 5.08	62.90 ± 34.15**
AKP (UI/I)	86.00 ± 21.40	87.00 ± 16.2
GGT (UI/I)	16.70 ± 10.70	93.22 ± 78.88*
Total bilirubin (μmol/L)	8.88 ± 4.23	13.22 ± 5.07
Conjugated bilirubin (μmol/L)	3.66 ± 2.16	4.18 ± 1.52

*p < 0.05, **p < 0.01 vs. healthy controls.

### Hepatic CCN1 was positively correlated with steatosis in patients with NASH

**Figure 2:**
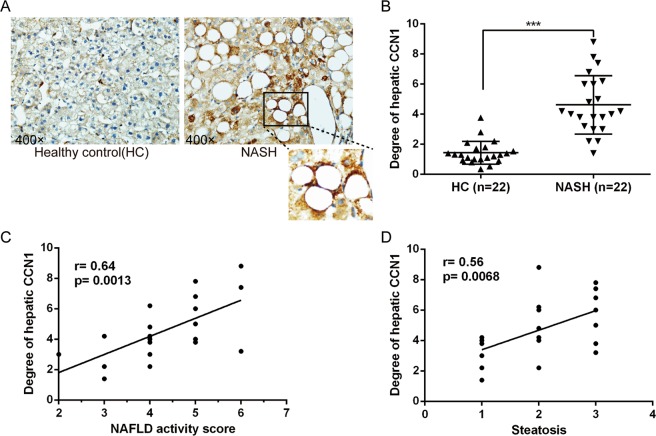
(**A**) Immunohistochemical staining of hepatic CCN1 in human livers from healthy controls and patients with NASH (400×). (**B**) Statistics of hepatic CCN1 expression in patients with NASH and healthy controls. (**C**) Hepatic CCN1 was positively associated with the NAFLD activity score (r = 0.64, p = 0.0013). (**D**) Hepatic CCN1 was positively associated with steatosis (r = 0.56, p = 0.0068). ***P < 0.001.

Immunohistochemical analysis showed that CCN1 was weakly expressed in the livers of healthy controls but strongly stained in steatotic livers of patients with NASH, especially in ballooning or fat laden hepatocytes in the centrolobular area (Fig. 2A,B). In order to explore the role of CCN1 in NASH, we analysed the relationship between CCN1 and the severity of NASH expressed by the NAFLD activity score (NAS), which comprises the grade of steatosis, lobular inflammation and balloon degeneration. We observed that the degrees of CCN1 expression in livers were positively associated with the NAS (r = 0.64, *P* = 0.0013) (Fig. 2C). Furthermore, the degree of CCN1 expression in livers was positively associated with steatosis (r = 0.56, p = 0.0068) (Fig. 2D) but was not associated with lobular inflammation and balloon degeneration.

### Overexpression of CCN1 aggravated the severity of MCD or HF-induced steatosis

**Figure 3:**
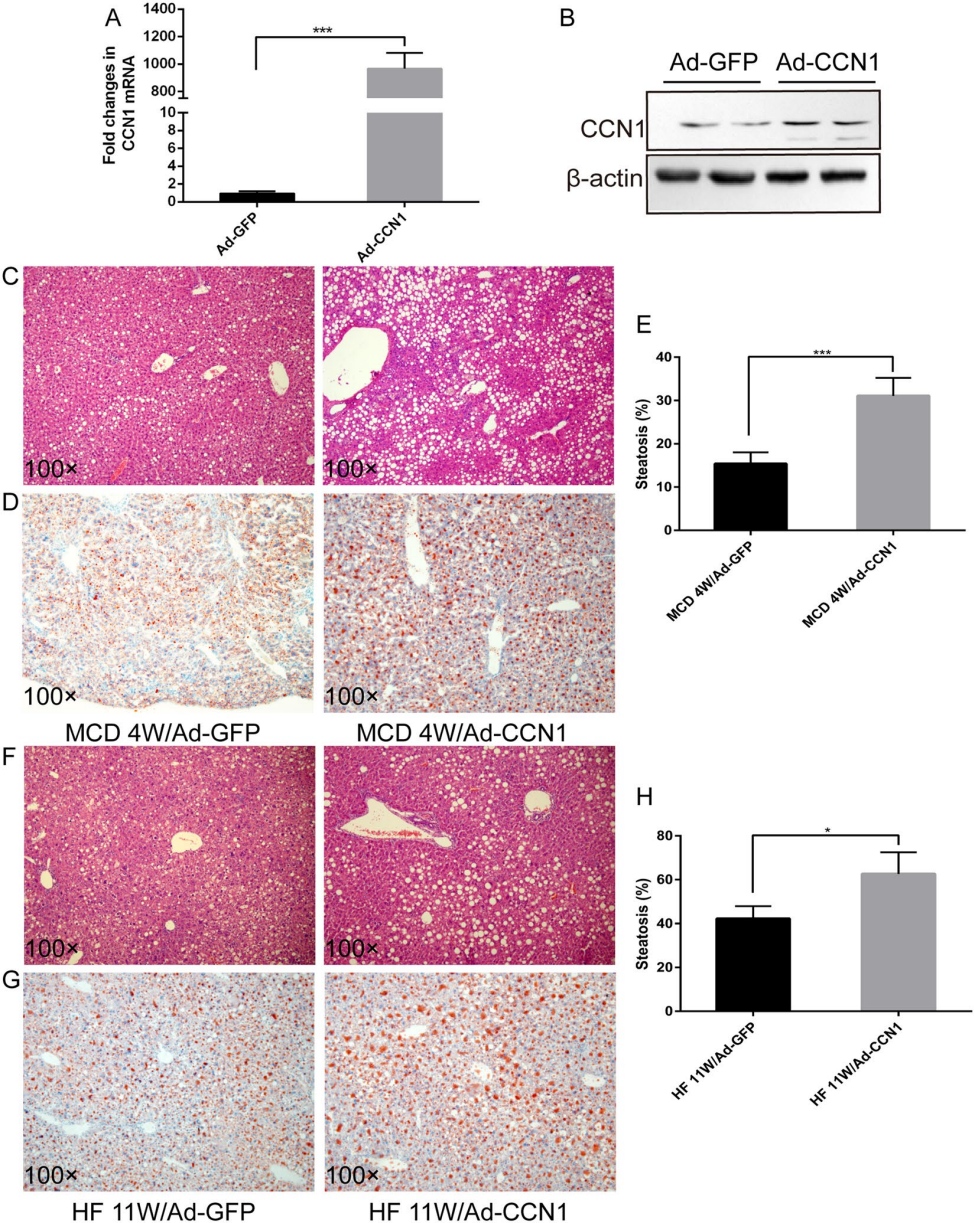
Upregulation of CCN1 aggravates MCD- or HF-induced hepatic steatosis. (**A**) Hepatic CCN1 mRNA was detected by qPCR in mice after Ad-GFP or Ad-CCN1 tail vein injection. (**B**) CCN1 protein was detected by western blotting in mice after Ad-GFP or Ad-CCN1 tail vein injection. (**C**) H&E staining of liver sections from MCD mice treated with Ad-GFP or Ad-CCN1. (**D**) Oil red O staining of liver sections from MCD mice treated with Ad-GFP or Ad-CCN1. (**E**) Statistical analysis of the area of steatosis in liver tissues from MCD mice treated with Ad-GFP or Ad-CCN1. (**F**) H&E staining of liver sections from HF mice treated with Ad-GFP or Ad-CCN1. (**G**) Oil red O staining of liver sections from HF mice treated with Ad-GFP or Ad-CCN1. (**H**) Statistical analysis of the area of steatosis in liver tissues from HF mice treated with Ad-GFP or Ad-CCN1. Statistical significance was assessed by two-tailed Student’s t test. The error bar indicates the SD. *P < 0.05; ***P < 0.001.

In order to demonstrate the role of CCN1 in the pathogenesis of steatosis, we controlled its expression by delivering adenoviruses (Ad) expressing GFP or CCN1 into mice through tail vein injection. After two weeks, we determined the CCN1 level in the liver by RT-PCR and western blotting and found that the CCN1 mRNA and protein levels were dramatically increased in the mice injected with Ad-CCN1 compared to the controls (Ad-GFP) (Fig. 3A,B). Mice fed a MCD diet were injected with Ad-CCN1 or Ad-GFP in week 2 and sacrificed in week 4. Consistently, haematoxylin and eosin (H&E) staining and oil red O staining showed a significant increase in hepatic steatosis in MCD/Ad-CCN1 mice (32% ± 4%) compared with MCD/Ad-GFP controls (15% ± 2%, Fig. 3C–E). To further evaluate the precise role of CCN1 in NASH, an additional HF-induced model was used. We applied a HF diet supplemented with cholesterol and cholate, which has been thought to induce pathological changes closely resembling human NAFLD. H&E staining and oil red O staining revealed that Ad-CCN1 mice developed more steatosis when fed a HF diet for 11 weeks (Fig. 3F–H). Together, these results suggest that overexpression of CCN1 increases the severity of steatosis.

### Gene expression profiling analysis in mice and murine primary hepatocytes treated with Ad-CCN1

**Figure 4:**
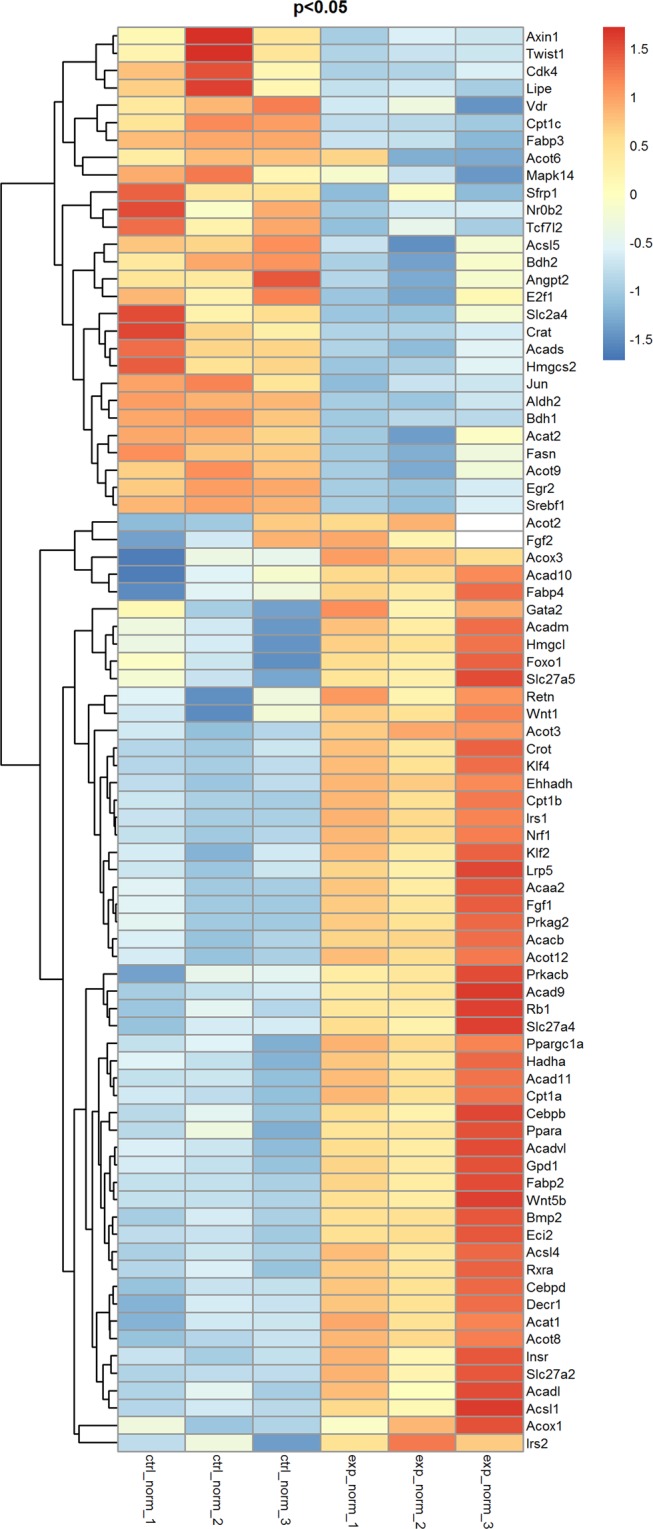
Heat map of the differentially expressed genes in the livers of MCD mice in the control and CCN1-overexpressing groups by microarray analyses (red: upregulated; green: downregulated).

In the mice fed with MCD, adenovirus expressing CCN1 or the control was administered in week 2, and the mice were sacrificed in week 4. Hepatic mRNAs were isolated from the two groups, including the Ad-control and Ad-CCN1 groups. The proportions of probes upregulated or downregulated significantly (fold changes ≥2) between the Ad-CCN1-1, 2, and 3 and Ad-control-1, 2, and 3 groups indicated that a variety of fatty acid metabolism-associated genes altered their expression levels in livers treated with Ad-CCN1. The heat map demonstrated distinguishable mRNA expression profiles between the two groups (Fig. 4). In total, separately and differentially expressed genes were identified between the experimental and control groups by microarray analysis. Of these genes, approximately 54 genes were upregulated (fold change ≥2), while 28 genes were downregulated (fold change <1).

**Figure 5:**
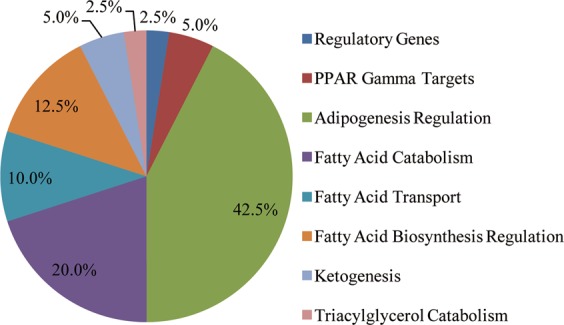
Taxonomic pie chart of fatty acid metabolism-associated genes in CCN1-overexpressing hepatocytes.

To assay gene changes in hepatocytes, we developed a 168-well gene expression array that contains genes representing the main fatty acid metabolism pathway. Overexpression of CCN1 was induced in primary murine hepatocytes to investigate the effect of CCN1 on fatty acid metabolism-associated genes. The gene expression profiles of fatty acid metabolism were detected by a qPCR-based array. In murine primary hepatocytes, we found that CCN1 overexpression significantly upregulated the expression level of fatty acid metabolism-associated genes, including adipogenesis regulation genes, fatty acid catabolism genes, fatty acid biosynthesis regulation genes, fatty acid transport genes, fatty acid regulatory genes, ketogenesis genes, PPAR gamma target genes, and triacylglycerol catabolism genes (Fig. 5), which was consistent with the qPCR-based analysis (Supplementary Table 1). According to the fold changes, the details, including 38 upregulated genes and 2 downregulated genes, are listed in Table 2.

**Table 2:** Genes significantly influenced in hepatocytes by overexpression of the CCN1 gene through microarray analysis.

No.	Gene Symbol	Description	*P* value	Fold-change	Regulation
1	Cfd	Complement factor D (adipsin) (Cfd), transcript variant 2	0.0015	0.3	down
2	Fabp5	Fatty acid binding protein 5, epidermal (Fabp5), transcript variant 2	0.0342	0.71	down
3	Sirt3	Sirtuin 3 (Sirt3), transcript variant 2	0.0455	1.81	up
4	Acacb	Acetyl-Coenzyme A carboxylase beta (Acacb)	0.0016	2.54	up
5	Cebpd	CCAAT/enhancer binding protein (C/EBP), delta (Cebpd)	0.044	1.97	up
6	Wnt5b	Wingless-type MMTV integration site family, member 5B (Wnt5b), transcript variant 2	0.0042	2.73	up
7	Cdkn1a	Cyclin-dependent kinase inhibitor 1 A (P21) (Cdkn1a), transcript variant 2	0.0003	3.4	up
8	Cdkn1b	Cyclin-dependent kinase inhibitor 1B (Cdkn1b)	0.0149	3.04	up
9	Ddit3	DNA-damage inducible transcript 3 (Ddit3), transcript variant 1	0	29.35	up
10	Hes1	Hairy and enhancer of split 1 (Drosophila) (Hes1)	0.0015	1.26	up
11	Sirt1	Sirtuin 1 (Sirt1), transcript variant 1	0.0074	2.05	up
12	Sirt2	Sirtuin 2 (Sirt2), transcript variant 2	0.007	1.29	up
13	Bmp2	Bone morphogenetic protein 2 (Bmp2)	0.0003	4.73	up
14	Klf4	Kruppel-like factor 4 (gut) (Klf4)	0.0048	3.26	up
15	Klf2	Kruppel-like factor 2 (lung) (Klf2)	0.007	1.82	up
16	Creb1	cAMP responsive element binding protein 1 (Creb1), transcript variant C	0.0299	1.79	up
17	Nrf1	Nuclear respiratory factor 1 (Nrf1), transcript variant 1	0.0022	1.68	up
18	Ppara	Rattus norvegicus peroxisome Proliferator activated receptor alpha (Ppara)	0.0225	1.64	up
19	Wnt5a	Wingless-type MMTV integration site family, member 5A (Wnt5a), transcript variant 2	0.0024	1.46	up
20	Ncoa2	Nuclear receptor coactivator 2 (Ncoa2), transcript variant 2	0.0199	1.52	up
21	Nr1h3	Nuclear receptor subfamily 1, group H, member 3 (Nr1h3), transcript variant 2	0.0111	1.4	up
22	Rb1	Retinoblastoma 1 (Rb1)	0.0067	1.92	up
23	Twist1	Twist homolog 1 (Drosophila) (Twist1)	0.0004	4.15	up
24	Acat1	Acetyl-Coenzyme A acetyltransferase 1 (Acat1)	0.0052	2.23	up
25	Acat2	Acetyl-Coenzyme A acetyltransferase 2 (Acat2)	0.0271	1.27	up
26	Ehhadh	Enoyl-Coenzyme A, hydratase/3-hydroxyacyl Coenzyme A dehydrogenase (Ehhadh)	0.0009	1.77	up
27	Acox2	Acyl-Coenzyme A oxidase 2, branched chain (Acox2), transcript variant 2	0.0008	2.18	up
28	Acsl6	Acyl-CoA synthetase long-chain family member 6 (Acsl6), transcript variant X1	0.0282	1.6	up
29	Acot2	Acyl-CoA thioesterase 2 (Acot2)	0.0062	3.02	up
30	Eci2	Enoyl-Coenzyme A delta isomerase 2 (Eci2), transcript variant 1	0.0316	1.17	up
31	Klf15	Kruppel-like factor 15 (Klf15)	0.0102	2.41	up
32	Slc27a3	Solute carrier family 27 (fatty acid transporter), member 3 (Slc27a3)	0.0001	5.86	up
33	Prkab1	Protein kinase, AMP-activated, beta 1 non-catalytic subunit (Prkab1)	0.0176	2.1	up
34	Prkab2	Protein kinase, AMP-activated, beta 2 non-catalytic subunit (Prkab2)	0.0317	1.34	up
35	Prkaca	Protein kinase, cAMP dependent, catalytic, alpha (Prkaca), transcript variant 2	0.037	1.61	up
36	Prkag2	Protein kinase, AMP-activated, gamma 2 non-catalytic subunit (Prkag2), transcript variant 2	0.0265	1.64	up
37	Prkag3	Protein kinase, AMP-activated, gamma 3 non-catalytic subunit (Prkag3)	0.0371	2.49	up
38	Hmgcl	3-Hydroxy-3-methylglutaryl-Coenzyme A lyase (Hmgcl)	0.0419	1.37	up
39	Oxct2a	3-Oxoacid CoA transferase 2A (Oxct2a)	0.012	2.43	up
40	Gpd2	Glycerol phosphate dehydrogenase 2, mitochondrial (Gpd2), transcript variant 1	0.0201	1.51	up

### Downregulation of CCN1 decreased the severity of MCD or HF-induced steatosis

**Figure 6:**
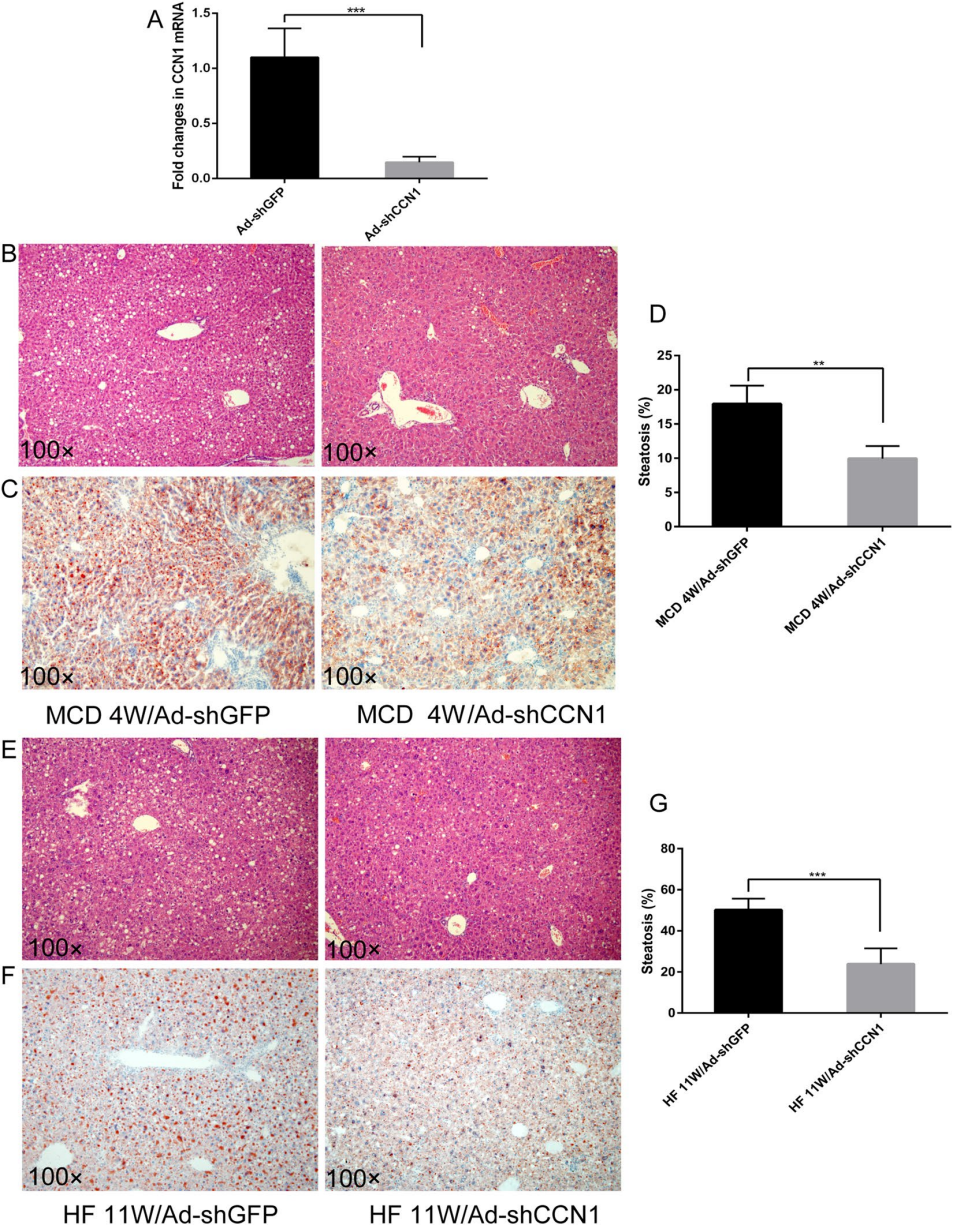
Downregulation of CCN1 alleviates MCD- or HF-induced hepatic steatosis. (**A**) Hepatic CCN1 mRNA was detected by qPCR in mice after Ad-shGFP or Ad-shCCN1 tail vein injection. (**B**) H&E staining of liver sections from MCD mice treated with Ad-shGFP or Ad-shCCN1. (**C**) Oil red O staining of liver sections from MCD mice treated with Ad-shGFP or Ad-shCCN1. (**D**) Statistical analysis of the area of steatosis in liver tissues from MCD mice treated with Ad-shGFP or Ad-shCCN1. (**E**) H&E staining of liver sections from HF mice treated with Ad-shGFP or Ad-shCCN1. (**F**) Oil red O staining of liver sections from HF mice treated with Ad-shGFP or Ad-shCCN1. (**G**) Statistical analysis of the area of steatosis in liver tissues from HF mice treated with Ad-shGFP or Ad-shCCN1. Statistical significance was assessed by two-tailed Student’s t test. The error bar indicates the SD. **P < 0.01; ***P < 0.001.

To confirm the potential utility of regulating CCN1 in treating murine steatohepatitis, recombinant adenovirus-mediated shGFP or shCCN1 was used to downregulate the expression of CCN1 in mice. The level of hepatic CCN1 was significantly decreased in mice injected with Ad-shCCN1 through the tail vein compared to the level in the control (Fig. 6A). In the mice fed the MCD diet, the adenovirus to decrease the expression of CCN1 was injected in week 2 after being fed the MCD diet, and the mice were sacrificed in week 4. Histological analysis revealed less lipid accumulation in the livers of Ad-shCCN1 mice fed the MCD diet than in the livers of Ad-shGFP mice (Fig. 6B–D). Moreover, reduced steatosis was also observed in Ad-shCCN1 mice fed a HF diet (Fig. 6E–G). These data suggested that targeting CCN1 may alleviate steatosis, which is the initiation of NASH.

### CCN1 exacerbates murine primary hepatocyte steatosis *in vitro*

**Figure 7:**
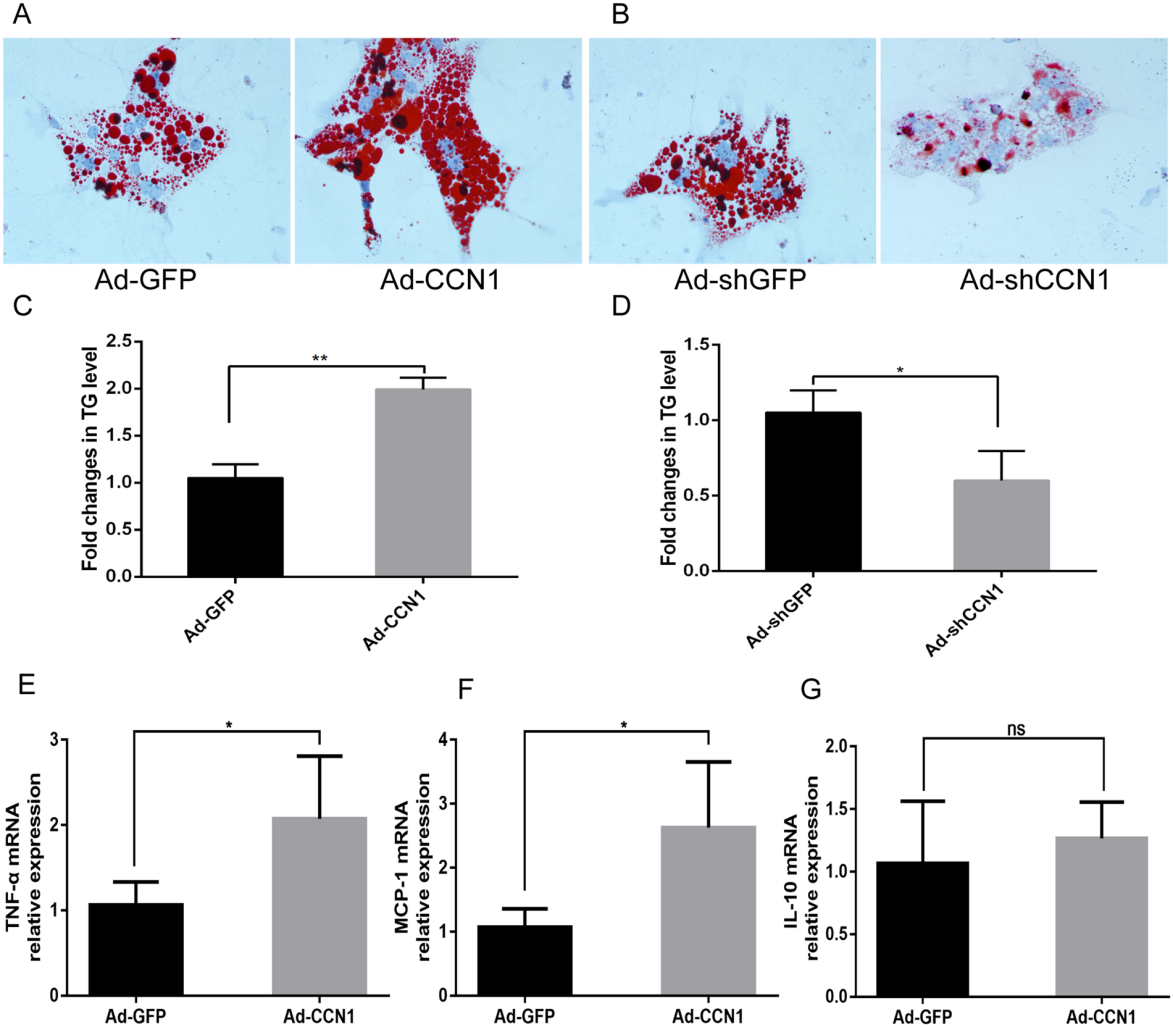
CCN1 exacerbates murine primary hepatocyte steatosis. (**A**) Oil red O-stained sections of primary hepatocytes treated with Ad-GFP or Ad-CCN1. (**B**) Statistical analysis of the TG concentration in murine primary hepatocytes treated with Ad-GFP or Ad-CCN1. (**C**) Oil red O-stained sections of primary hepatocytes treated with Ad-shGFP or Ad-shCCN1. (**D**) Statistical analysis of the TG concentration in murine primary hepatocytes treated with Ad-shGFP or Ad-shCCN1. (**E**) Expression of TNF-α was quantified in primary hepatocytes of Ad-GFP group and Ad-CCN1group. (**F**) Expression of MCP-1 was quantified in primary hepatocytes of Ad-GFP group and Ad-CCN1group. (**G**) Expression of IL-10 was quantified in primary hepatocytes of Ad-GFP group and Ad-CCN1 group. Statistical significance was assessed by two-tailed Student’s t test. The error bar indicates the SD. *P < 0.05; **P < 0.01; ns: no significance.

In our previous study, we found that CCN1 promotes inflammation in a murine NAFLD model^18^. In order to determine if the effect of CCN1 in inducing steatosis depended on promoting inflammation, we isolated and cultured murine primary hepatocytes *in vitro*. Primary hepatocytes were exposed to FFAs (oleic/palmitic 2:1 molar ratio) for 24 h to induce hepatic lipid accumulation. The content of intracellular lipid droplets was determined by oil red O staining. Primary murine hepatocytes were infected by adenovirus controlling the expression of CCN1 24 h before administration of FFAs. Oil red O staining showed that overexpression of CCN1 increased lipid accumulation in primary murine hepatocytes (Fig. 7A). In contrast, downregulation of CCN1 decreased lipid accumulation (Fig. 7B). Further study confirmed that overexpression of CCN1 increased the TG content compared with the Ad-GFP-treated group in primary murine hepatocytes, while downregulation of CCN1 reduced the TG content (Fig. 7C,D). Overexpression of CCN1 increased hepatic production of pro-inflammatory cytokines, including tumor necrosis factor alpha (TNF-α) and monocyte chemoattractant protein-1 (MCP-1) compared to the controls (Fig. 7E,F). There was no significant difference in the expression of interleukin (IL)-10 between Ad-CCN1-treated and Ad-GFP-treated samples (Fig. 7G).

### CCN1 induces severe hepatic inflammation in NASH

**Figure 8:**
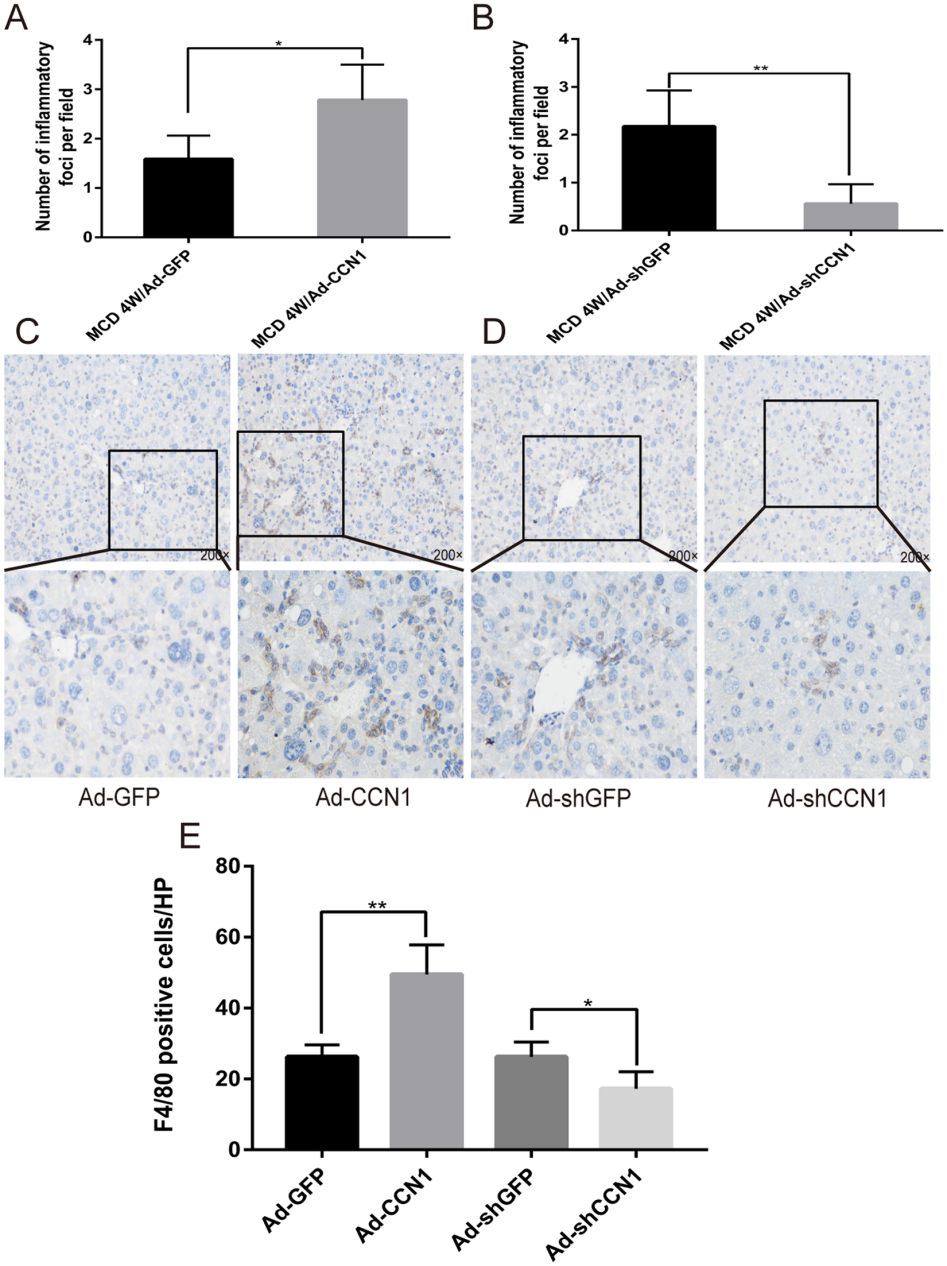
CCN1 aggravates hepatic inflammation and promotes macrophage infiltration during NASH development. (**A**) Statistical analysis of inflammatory foci in liver tissues from MCD mice treated with Ad-GFP or Ad-CCN1. (**B**) Statistical analysis of the area of steatosis in liver tissues from MCD mice treated with Ad-shGFP or Ad-shCCN1. (**C,D**) Representative F4/80 immunohistochemistry from MCD mice treated with Ad-GFP, Ad-CCN1, Ad-shGFP or Ad-shCCN1. (**E**) Quantification of F4/80-positive cells is shown as the total number of positive cells per field. Statistical significance was assessed by two-tailed Student’s t test. The error bar indicates the SD. *P < 0.05; **P < 0.01.

Macrophages play a very important role in initiating inflammation in NASH, and we proved that CCN1 overexpression by adenovirus could increase hepatic inflammation (Fig. 8A,B). Herein, we examined the staining and expression of F4/80, a well-established histological marker of murine macrophages, to assess the hepatic infiltration of macrophages during NASH development. The number of F4/80-positive cells was obviously increased in the livers of MCD/Ad-CCN1 mice compared with the livers of MCD/Ad-GFP mice. In contrast, the number of F4/80-positive cells was decreased in the livers of MCD/Ad-shCCN1 mice compared with the livers of MCD/Ad-shGFP mice (Fig. 8C–E).

### CCN1 overexpression increases the expression of apoptosis-related proteins in murine hepatocytes

**Figure 9:**
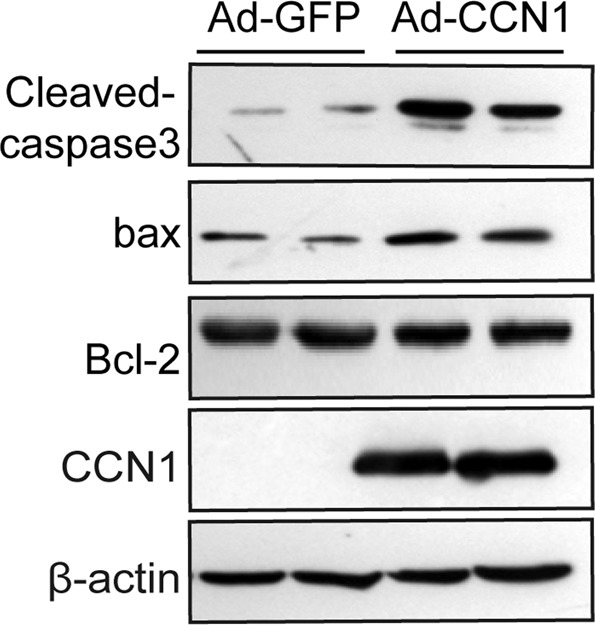
Apoptotic proteins were detected by western blot.

We performed western blot assays to assess the expression levels of apoptosis- associated proteins (cleaved-caspase 3, Bax and Bcl-2) to further elucidate the molecular mechanisms by which CCN1 participates in NASH (Fig. 9). The results showed that CCN1 overexpression increased the expression of cleaved-caspase 3 and the pro-apoptotic protein Bax. There was no significant difference in the level of the anti-apoptotic protein Bcl-2.

## Discussion

NAFLD is a genetic, environmental, metabolic and stress-associated liver disease without a history of excessive drinking and is characterized by steatosis and fat storage in liver parenchymal cells^2,20,21^. NAFLD is often associated with obesity and is considered a benign condition. However, hepatic steatosis can progress to the clinical state of NASH, which is a precursor to more serious liver diseases, such as cirrhosis and hepatocellular carcinoma^1^. Apoptosis is the main pathogenic feature of NASH^22^. Inflammation is also a remarkable feature in NASH. The widely used animal models of NASH with histological characteristics that most closely resemble those seen in humans are the MCD and HF dietary mouse models. The advantage of the MCD model is that it can induce steatohepatitis rapidly. However, the MCD model shows severe body weight loss and does not develop insulin resistance. Compared with other NASH models, the MCD model can cause more severe inflammation, oxidative stress and apoptosis. Fat accumulation in the form of triglycerides in hepatocytes is the major feature of hepatic steatosis^23^. FFA-overloaded murine primary hepatocytes exhibited comparable lipid contents, which was similar to lipid accumulation in hepatocytes obtained from human steatotic liver.

The members of CCN family proteins includes CCN1, CCN2, CCN3, CCN4, CCN5, and CCN6^8^. They share four conserved functional domains and play important roles in bone development, wound healing, fibrosis, and cancer^8,24–26^. The abnormal expression of certain CCNs is associated with disease progression and poor prognosis^24^. For example, CCN2 has been demonstrated to induce chondrocyte proliferation through the MAPK/ERK signalling pathway, and down-regulation of CCN2 expression inhibits cell proliferation and increases cell apoptosis^27^. The expression of CCN3 in adipose tissue could play a key role in the development of obesity^28^. A study revealed that CCN3 expression is closely related to the formation of fat mass and the development of obesity^28^. CCN4 has been linked to neurodegenerative protection^25^. CCN5 is an important negative regulator of motility through matrix metalloproteinase-2 gene expression modulation^29^. Finally, CCN6 is considered a tumour suppressor for breast cancer^30^.

In this study, we observed a close relationship between upregulated CCN1 and NASH that was confirmed in MCD or HF-induced NAFLD mice. We determined the function of CCN1 in NAFLD by using gain-of-function and loss-of-function mouse models. Here, we showed that CCN1 upregulation was associated with fatty livers and that CCN1 has a positive regulating effect on hepatic steatosis and the inflammatory response in NASH. Furthermore, we found that CCN1 overexpression significantly upregulated the expression of fatty acid metabolism-associated genes using a qPCR-based array in primary hepatocytes. The F4/80 results revealed that CCN1 promotes macrophage infiltration, and similar results were found in our previous studies^18^, except with different experimental methods. We assessed the expression levels of cleaved-caspase 3 and the pro-apoptotic protein Bax. Western blot assays showed that CCN1 overexpression upregulated cleaved-caspase 3 and Bax in primary hepatocytes. In our study, we demonstrated that CCN1 could regulate lipometabolism and promote hepatocyte apoptosis in the pathogenesis of NASH and could drive macrophage infiltration into the liver, which was consistent with previous studies. In our human study, we showed that CCN1 was increased in liver tissues and serum of patients with NASH compared with healthy controls. Most importantly, hepatic CCN1 is positively correlated with NAS and steatosis. Taken together, our data suggest that CCN1 plays a key role in the pathogenesis of steatohepatitis. These findings open up new insight into the treatment of NASH in humans.

In summary, we explored the detailed mechanism by which CCN1 participates in the pathogenesis of NAFLD and showed for the first time that CCN1 may be involved in the pathogenesis of human NASH. CCN1 may become a potential therapeutic target of human NASH in the future.

## Materials and Methods

### Patient samples

Fresh frozen specimens of NASH tissues and healthy specimens were collected in our research. The definition of NASH was confirmed by liver biopsy. Healthy specimens were obtained from patients who had undergone liver resection for a liver haemangioma. The detailed clinical characteristics of the patients are listed in Table 1. In addition, serum samples from 22 patients with NASH and 22 healthy controls were also obtained and stored at −80 °C for further analysis. All the patients participating signed an informed consent form, and the study was approved by the ethical committee of Nantong Third People’s Hospital, Nantong University. All experiments were performed in accordance with relevant guidelines and regulations of the hospital.

### Adenoviral infection

Adenoviruses (Ad) expressing CCN1 (Ad-CCN1 and Ad-shCCN1) were constructed by Oobio Biotechnology (Shanghai, China). C57BL/6 mice were infected with GFP or CCN1 adenoviruses (2 × 10^9^ particles/mouse) via tail vein injection. Two weeks post-injection, the mice were sacrificed, and liver tissues were harvested for further experiments.

### Animal models

The experiments were performed in mice on a C57BL/6 background because this strain is widely used to study diet-induced obesity models. Eight-week-old male C57BL/6 mice were purchased from Shanghai SLAC Laboratory Animal Central (Shanghai, China) and allowed to acclimatize for one week before being fed specific diets. Mice were housed in a pathogen-free animal facility at 22 °C under a controlled 12-h light/dark cycle. Mice were given free access to standard chow and tap water. C57BL/6 mice were fed methionine and choline deficient (MCD) (Research Diets, Inc., New Brunswick, NJ, USA) or control diets to induce steatohepatitis. A high-fat (HF) diet model is also widely used for the basic study of NASH because it causes obesity and insulin resistance. C57BL/6 mice were fed a HF diet (60 kcal% fat, Research Diets, Inc.) or control diets to lead to steatohepatitis. All animal care protocols and experimental procedures were approved by the Animal Experimental Ethics Committee of Nantong University and performed in accordance with relevant guidelines and regulations.

### ELISA for serum CCN1 analysis

The human or murine serum level of CCN1 was detected by the DRG CCN1 ELISA kit (DRG Instruments GmbH, Germany) and Xitang CCN1 ELISA kit (Shanghai Xitang Biotechnology, Shanghai, China) following the manufacturer’s instructions, respectively.

### qRT-PCR analysis

Total RNA from tissues was extracted by using TRIzol reagent (TakaraBIO, Dalian, China). RNA purity and concentration were determined by a NanoDrop ND-2000 spectrophotometer (Thermo Fisher Scientific, Waltham, MA, USA). RNA was reverse transcribed using a Prime ScriptTM RT Reagent Kit (Takara Bio, Dalian, China) according to the manufacturer’s protocol. The reactions were performed in a Bio-Rad Real-Time PCR Detection System using SYBR® Green Master Mix (Vazyme Biotech, Nanjing, China). The primers used for the real-time PCR reaction were as follows: human CCN1: forward, 5′-CTGAGTGTATGCCATTCGG-3′, reverse, 5′-ACTGCTGTATCCCAATAAG-3′; mouse CCN1: forward, 5′-GTGCCGCCTGGTGAAAGAGA-3′, reverse, 5′-GCTGCATTTCTTGCCCTTTTT

TAG-3′; human β-actin: forward, 5′-GGACTTCGAGCAAGAGATGG-3′, reverse, 5′-AGGAAGGAAGGCTGGAAGAG-3′; mouse β-actin: forward, 5′-CTAAGGCC

AACCGTGAAAAG-3′, reverse, 5′-GGTACGACCAGAGGCATACA-3′. After a pre-denaturation step at 95 °C for 5 min, 40 cycles of PCR were performed as follows: 10 s denaturation at 95 °C and 30 s annealing at 60 °C. The fold amplification for each gene was calculated by using the comparative 2^−ΔΔCt^ method.

### Western blotting

Tissues were lysed with RIPA buffer containing protease inhibitors (Beyotime, Shanghai, China). The protein concentrations were detected by using a BCA Protein Assay Kit (Beyotime). Protein extracts were separated by 10% SDS-PAGE and then electrophoretically transferred to PVDF membranes. Immunoblots were reacted with primary antibodies against human CCN1 (Abcam, Cambridge, MA, United States) or β-actin (Santa Cruz, CA, USA), followed by detection with horseradish peroxidase (HRP)-conjugated secondary antibodies (MR Biotech, Shanghai, China). The signal was examined using an ECL system (Thermo Fisher Scientific).

### Immunohistochemistry

The paraffin-embedded tissue samples were sectioned into 4-μm slices and embedded with paraffin after fixation with 10% formaldehyde. After deparaffinization, the slices were incubated with 3% H_2_O_2_ for 15 min, and antigen was retrieved by boiling the sections in citrate buffer (pH 6.0) in a microwave for 10 min. After cooling, 10% goat serum was used to block any non-specific reactions for 30 min at room temperature. The sections were then incubated with CCN1 primary antibodies (1:100) or anti-F4/80 antibody (1:50, Abcam, Cambridge, MA, United States) overnight at 4 °C. After rinsing with PBS, the sections were incubated with an HRP-conjugated secondary antibody (Shanghai Long Island Biotec., Shanghai, China) for 30 min at room temperature. PBS was then used to rinse the specimens, and 3,3′-diaminobenzidine (DAB, Maixin-Bio, Guangzhou, China) was applied for 60 s. Haematoxylin was applied for nuclear staining, and the stained sections were cover-slipped for imaging by light microscopy (Olympus, Japan). The severity of liver disease was evaluated histologically on HE-stained sections according to the NAFLD activity score by a liver pathologist. Five high-power fields were randomly chosen for assessment in every sample. The intensity of CCN1 deposition was scored semiquantitatively on a 0–3-point scale, and the area of CCN1 deposition was scored on a 0–4-point scale. The intensity score multiplied by the area score was the CCN1 degree.

### Murine primary hepatocyte isolation and culture

Primary hepatocytes were isolated from 6-week-old male C57BL/6 mice via liver perfusion as previously described. Briefly, livers were perfused through the portal vein with calcium and magnesium-free Hanks’s balanced salt solution (HBSS, Invitrogen, Carlsbad, CA, USA) followed by Dulbecco’s modified Eagle’s medium (DMEM, Invitrogen) containing collagenase (collagenase types I and IV; Sigma). Primary hepatocytes were separated via centrifugation at 50 *g* for 5 min. The obtained hepatocytes were re-suspended in DMEM. Trypan blue exclusion assays indicated that cell viability was > 95%. Hepatocytes were cultured in DMEM supplemented with 10% foetal bovine serum, 100 U/ml penicillin and 100 mg/ml streptomycin in a 5% CO2/water-saturated incubator at 37 °C. Free fatty acids (FFAs, mixture of oleic acid and palmitic acid at a ratio of 2:1; Sigma) were added to the medium for 24 h to establish the *in vitro* model of lipid accumulation in hepatocytes.

### PCR arrays

Total RNA was extracted with TRIzol (Invitrogen) and quantified by a NanoDrop Spectrophotometer (NanoDrop Technologies). Reverse transcription was performed with 500 ng of total RNA using an RT^2^ first-strand kit (SABiosciences, Frederick, MD, USA). Analysis was performed using RT^2^ Profile PCR Arrays (Qiagen, USA). The array profiles the expression of 168 key genes involved in the fatty acid metabolism pathway in the liver.

### Histology

Mouse liver samples were fixed in 10% formaldehyde and processed for haematoxylin and eosin (H&E) and oil red O staining by standard methods. The severity of liver disease in mice was evaluated histologically on H&E-stained sections by a histopathologist. Liver tissues were scored for steatosis and inflammation. The degree of steatosis was scored as the percentage of hepatocytes containing lipid droplets. Hepatocellular inflammation was scored as 0 to 3 according to the severity: 0, no inflammatory foci per ×10 field; 1, 1–2 inflammatory foci per ×10 field; 2, 3–4 inflammatory foci per ×10 field; and 3, >4 inflammatory foci per ×10 field.

### Concentration of triglycerides in hepatocytes

To quantify hepatic triglyceride content, murine primary hepatocytes were lysed with buffer from an enzymatic kit (TG, Nanjing Jiancheng Bioengineering Institute, Nanjing, China) according to the manufacturer’s instructions, and the concentration of triglyceride was adjusted to the total protein.

### Statistical analysis

All data are expressed as the mean ± standard deviation (SD). All statistical analyses were performed using GraphPad Prism software version 6.0 (GraphPad, Inc., La Jolla, CA, USA). The significance of differences was determined by Student’s t test, and a *P* value less than 0.05 was considered significant.

## Supplementary information

Supplementary Table 1.
