# Supplementary material for: CCN1 promotes hepatic steatosis and inflammation in non-alcoholic steatohepatitis
Source: Sci Rep. 2020 Feb 21;10:3201. doi: 10.1038/s41598-020-60138-8 (PMC7035350; doi:10.1038/s41598-020-60138-8)
Supplement: Supplementary file 1 — Supplementary Table 1. [file 41598_2020_60138_MOESM1_ESM.pdf]

CCN1 promotes hepatic steatosis and inflammation in non-alcoholic steatohepatitis

Linling Ju[1,*], Yan Sun[2,*], Hong Xue[3], Lin Chen[1], Chunyan Gu[4], Jianguo Shao[1], Rujian Lu[5], Xi Luo[1], Jue Wei[6], Xiong Ma[7], Zhaolian Bian[1]

Supplementary Table 1: Respective pathway genes included in the fatty acid metabolism PCR array.

Pathway	Gene
**Regulatory Genes**	Adipoq, Cfd, Lep, Retn, Agt, Angpt2,Lipe, Lpl
**PPAR Gamma Targets**	Ipoq, Cebpa, Cfd, Fabp4, Fasn, Irs2, Klf15,Pparg, Ppargc1a (PGC-1a), Retn, Sirt3, Srebf1
**Adipogenesis Regulation**	
Pro-Adipogenesis	Acacb, Axin1, Ccnd1, Cdk4, Cebpb, Cebpd, Dkk1, E2f1, Fabp4, Fasn, Fgf1, Fgf2, Irs2, Jun, Lmna, Rxra, Sfrp1, Sfrp5, Slc2a4 (GLUT4), Wnt5b
Anti-Adipogenesis	Adrb2, Cdkn1a, Cdkn1b, Ddit3, Dlk1, Foxo1, Hes1, Lrp5, Ncor2, Runx1t1, Shh, Sirt1, Sirt2, Taz, Tcf7l2, Tsc22d3, Vdr, Wnt1, Wnt3a
Pro-White Adipose Tissue	Bmp2, Bmp4, Cebpa, Egr2, Fgf10, Klf15, Klf4
Anti-White Adipose Tissue	Gata2, Gata3, Klf2, Klf3
Pro-Brown Adipose Tissue	Bmp7, Creb1, Dio2, Foxc2, Insr, Irs1, Mapk14, Nrf1, Ppara, Ppard, Prdm16, Sirt3, Src, Ucp1, Wnt5a
Anti-Brown Adipose Tissue	Ncoa2, Nr0b2, Nr1h3, Rb1, Twist1, Wnt10b
**Fatty Acid Catabolism**	
Acetyl-CoA Transferases	Acaa1a, Acaa2, Acat1, Acat2
Acyl-CoA Dehydrogenases	Acad9, Acad10, Acad11, Acadl, Acadm, Acads, Acadsb, Acadvl, Ehhadh, Gcdh
Acyl-CoA Oxidases	Acox1, Acox2, Acox3
Acyl-CoA Synthetases	Acsbg1, Acsbg2, Acsl1, Acsl3, Acsl4, Acsl5, Acsl6, Acsm2, Acsm3, Acsm4, Acsm5
Acyl-CoA Thioesterases	Acot2, Acot3, Acot6, Acot7, Acot8, Acot9, Acot12
Carnitine Transferases	Cpt1a, Cpt1b, Cpt1c, Cpt2, Crat, Crot
Other	Aldh2, Decr1, Decr2, Echs1, Hadha, Mcee, Mut, Eci2, Pecr, Ppa1
**Fatty Acid Transport**	Cpt1a, Cpt1b, Cpt1c, Cpt2, Crat, Crot, Fabp1, Fabp2, Fabp3, Fabp4, Fabp5, Fabp6, Slc27a1, Slc27a2, Slc27a3, Slc27a4, Slc27a5, Slc27a6

**Fatty Acid Biosynthesis Regulation**	Prkaa1, Prkaa2, Prkab1, Prkab2, Prkaca, Prkacb, Prkag1, Prkag2, Prkag3
**Ketogenesis**	Bdh1, Bdh2, Hmgcl, Hmgcs1, Hmgcs2, Oxct2a
**Triacylglycerol Catabolism**	Gk2, Gpd1, Gpd2, Gyk, Lipe, Lpl
